# Supplementary material for: Comparative genomics provides new insights into the diversity, physiology, and sexuality of the only industrially exploited tremellomycete: Phaffia rhodozyma
Source: BMC Genomics. 2016 Nov 9;17:901. doi: 10.1186/s12864-016-3244-7 (PMC5103461; doi:10.1186/s12864-016-3244-7)
Supplement: Additional file 6: — List of orphan genes with links to PFAM (related to Additional file 1: Table S1). (ZIP 1428 kb) [file 12864_2016_3244_MOESM6_ESM.zip › BLAST_HTML_FTR/G05503_P.html]

BLAST Search Results


```
BLASTP 2.2.27+


Reference:
Stephen F. Altschul, Thomas L. Madden, Alejandro A. Schäffer,
Jinghui Zhang, Zheng Zhang, Webb Miller, and David J. Lipman (1997),
"Gapped BLAST and PSI-BLAST: a new generation of protein database
search programs", Nucleic Acids Res. 25:3389-3402.


Reference for
composition-based statistics:
Alejandro A. Schäffer, L. Aravind, Thomas L. Madden, Sergei
Shavirin, John L. Spouge, Yuri I. Wolf, Eugene V. Koonin, and
Stephen F. Altschul (2001), "Improving the accuracy of PSI-BLAST
protein database searches with composition-based statistics and
other refinements", Nucleic Acids Res. 29:2994-3005.


Database: nr
           71,551,133 sequences; 26,053,659,533 total letters


Query= G05503_P

Length=295
                                                                      Score     E
Sequences producing significant alignments:                          (Bits)  Value

emb|CED83394.1|  hypothetical protein [Xanthophyllomyces dendrorh...   591    0.0  
gb|KLO83362.1|  uncharacterized protein LW93_15032 [Fusarium fuji...  41.6    0.96 
emb|CCT69232.1|  uncharacterized protein FFUJ_14268 [Fusarium fuj...  40.8    1.4  
gb|KLP19063.1|  uncharacterized protein LW94_1085 [Fusarium fujik...  40.4    1.8  
ref|WP_019069892.1|  oxidoreductase [Streptomyces sp. R1-NS-10]       38.9    4.9  
ref|XP_004222223.1|  hypothetical protein PCYB_084370 [Plasmodium...  39.3    5.1  


 >emb|CED83394.1| hypothetical protein [Xanthophyllomyces dendrorhous]
Length=294

 Score =  591 bits (1524),  Expect = 0.0, Method: Compositional matrix adjust.
 Identities = 294/294 (100%), Positives = 294/294 (100%), Gaps = 0/294 (0%)

Query  1    MSIPTERPFRKFQSEAWNMGSTVHVNSANLIDPELQSALASVGERVRNSIVKSNPTVATD  60
            MSIPTERPFRKFQSEAWNMGSTVHVNSANLIDPELQSALASVGERVRNSIVKSNPTVATD
Sbjct  1    MSIPTERPFRKFQSEAWNMGSTVHVNSANLIDPELQSALASVGERVRNSIVKSNPTVATD  60

Query  61   PVRKNVPTRTFSRAASTMNYPIVSDPFAASSRSTSGFARSFSQYIPARSFAPPAEEEDDS  120
            PVRKNVPTRTFSRAASTMNYPIVSDPFAASSRSTSGFARSFSQYIPARSFAPPAEEEDDS
Sbjct  61   PVRKNVPTRTFSRAASTMNYPIVSDPFAASSRSTSGFARSFSQYIPARSFAPPAEEEDDS  120

Query  121  FANGIIEGDKEAPAFGEGELGWGDGDAAETNGELFGGERLGAISGDRIHEDDDESICSPA  180
            FANGIIEGDKEAPAFGEGELGWGDGDAAETNGELFGGERLGAISGDRIHEDDDESICSPA
Sbjct  121  FANGIIEGDKEAPAFGEGELGWGDGDAAETNGELFGGERLGAISGDRIHEDDDESICSPA  180

Query  181  PSLSPPGSPSRTLEPQISSSSPLPPANSASLSKTRSFKTVPSFVESRHNMLSNLRAQTFS  240
            PSLSPPGSPSRTLEPQISSSSPLPPANSASLSKTRSFKTVPSFVESRHNMLSNLRAQTFS
Sbjct  181  PSLSPPGSPSRTLEPQISSSSPLPPANSASLSKTRSFKTVPSFVESRHNMLSNLRAQTFS  240

Query  241  SPEVDPEEAETFRRHASAGWREDLIPAEKGRLESKPEEPWKDLNLHSFANVEDF  294
            SPEVDPEEAETFRRHASAGWREDLIPAEKGRLESKPEEPWKDLNLHSFANVEDF
Sbjct  241  SPEVDPEEAETFRRHASAGWREDLIPAEKGRLESKPEEPWKDLNLHSFANVEDF  294


>gb|KLO83362.1| uncharacterized protein LW93_15032 [Fusarium fujikuroi]
Length=673

 Score = 41.6 bits (96),  Expect = 0.96, Method: Compositional matrix adjust.
 Identities = 40/114 (35%), Positives = 53/114 (46%), Gaps = 15/114 (13%)

Query  112  PPAEEEDDSFANGIIEGDKEA-PAFGEGELGWGDGDAAETNGELFGGERLGAISGDRIHE  170
            P A E   S++  ++E + E+ PA+    L  GD D    +GE+       A S      
Sbjct  534  PKAPESQSSYSRSVVESEPESEPAWS---LPSGDEDTNTLDGEMTPLPSPSATSSTIFTP  590

Query  171  DDDESICSPAPSLSPPGSPSRTLEPQISSSSPL----------PPANSASLSKT  214
             D+ S  SP PSLSPPG P+  L P   SS+PL          PP NSA+  K 
Sbjct  591  TDNRSTRSPVPSLSPPG-PAPALRPVSPSSTPLSTGIFLFRGIPPTNSAAKFKC  643


>emb|CCT69232.1| uncharacterized protein FFUJ_14268 [Fusarium fujikuroi IMI 58289]
 gb|KLP06735.1| uncharacterized protein Y057_11411 [Fusarium fujikuroi]
Length=673

 Score = 40.8 bits (94),  Expect = 1.4, Method: Compositional matrix adjust.
 Identities = 40/114 (35%), Positives = 53/114 (46%), Gaps = 15/114 (13%)

Query  112  PPAEEEDDSFANGIIEGDKEA-PAFGEGELGWGDGDAAETNGELFGGERLGAISGDRIHE  170
            P A E   S++  ++E + E+ PA+    L  GD D    +GE+       A S      
Sbjct  534  PKAPESQSSYSRSVVESEPESEPAWS---LPSGDEDTNTLDGEVTPLPSPSATSSTIFTP  590

Query  171  DDDESICSPAPSLSPPGSPSRTLEPQISSSSPL----------PPANSASLSKT  214
             D+ S  SP PSLSPPG P+  L P   SS+PL          PP NSA+  K 
Sbjct  591  TDNRSTRSPVPSLSPPG-PAPALRPVSPSSTPLSTGIFLFRGIPPTNSAAKFKC  643


>gb|KLP19063.1| uncharacterized protein LW94_1085 [Fusarium fujikuroi]
Length=673

 Score = 40.4 bits (93),  Expect = 1.8, Method: Compositional matrix adjust.
 Identities = 40/114 (35%), Positives = 53/114 (46%), Gaps = 15/114 (13%)

Query  112  PPAEEEDDSFANGIIEGDKEA-PAFGEGELGWGDGDAAETNGELFGGERLGAISGDRIHE  170
            P A E   S++  ++E + E+ PA+    L  GD D    +GE+       A S      
Sbjct  534  PKAPESQSSYSRSLVESEPESEPAWS---LPSGDEDTNTLDGEVTPLPSPSATSSTIFTP  590

Query  171  DDDESICSPAPSLSPPGSPSRTLEPQISSSSPL----------PPANSASLSKT  214
             D+ S  SP PSLSPPG P+  L P   SS+PL          PP NSA+  K 
Sbjct  591  TDNRSTRSPVPSLSPPG-PAPALRPVSPSSTPLSTGIFLFRGIPPTNSAAKFKC  643


>ref|WP_019069892.1| oxidoreductase [Streptomyces sp. R1-NS-10]
Length=311

 Score = 38.9 bits (89),  Expect = 4.9, Method: Compositional matrix adjust.
 Identities = 33/111 (30%), Positives = 53/111 (48%), Gaps = 12/111 (11%)

Query  100  SFSQYIPARSFAPPAEEEDDS-FANGIIEGDKEAPAFGEGELGWGDG------DAAETNG  152
            ++++  P RS    A ++  S   +G +E + E PA  E    W +       D   T+G
Sbjct  176  AWTRLTPWRSMLAAALDQAKSKITSGAVESEAENPA-AELLARWLEARLKVKIDRVVTDG  234

Query  153  ELFGGERLGAISGDRIHEDDDESICSPAPSLSPPGSPSRTLEPQISSSSPL  203
             +    RLG  +GD + +  D     P  +LS PG PSRTL  ++ ++S L
Sbjct  235  PVITAVRLGTGAGDIVIDRPD----GPLATLSLPGQPSRTLALKVRTTSEL  281


>ref|XP_004222223.1| hypothetical protein PCYB_084370 [Plasmodium cynomolgi strain 
B]
 dbj|GAB66276.1| hypothetical protein PCYB_084370 [Plasmodium cynomolgi strain 
B]
Length=2550

 Score = 39.3 bits (90),  Expect = 5.1, Method: Composition-based stats.
 Identities = 25/84 (30%), Positives = 43/84 (51%), Gaps = 7/84 (8%)

Query  138   GELGWGDGDAAETNGELFGGERLGAISGDRIHEDDDESICSPAPSLSPP-----GSPSRT  192
             GE+G G GD+A +      G     +S + + +D   S  +PA S +P       +P + 
Sbjct  1546  GEIGTGAGDSAISAVSAVSGTNNANVS-NGVSDDQAASSANPANSANPANTANSATPDKA  1604

Query  193   LEPQISSSSPLPPANSASLSKTRS  216
               P  +S++P PPA+S +L K ++
Sbjct  1605  APPD-NSANPAPPAHSGALQKEKN  1627


Lambda      K        H        a         alpha
   0.312    0.128    0.375    0.792     4.96 

Gapped
Lambda      K        H        a         alpha    sigma
   0.267   0.0410    0.140     1.90     42.6     43.6 

Effective search space used: 2247310944536


  Database: nr
    Posted date:  Sep 23, 2015 12:05 AM
  Number of letters in database: 26,053,659,533
  Number of sequences in database:  71,551,133


Matrix: BLOSUM62
Gap Penalties: Existence: 11, Extension: 1
Neighboring words threshold: 11
Window for multiple hits: 40
```
